# Isolation of High-Molecular-Weight DNA for Long-Read Sequencing Using a High-Salt Gel Electroelution Trap

**DOI:** 10.1021/acs.analchem.3c03894

**Published:** 2023-11-22

**Authors:** Ruslan Kalendar, Konstantin I. Ivanov, Olga Samuilova, Ulykbek Kairov, Andrey A. Zamyatnin

**Affiliations:** † Institute of Biotechnology, Helsinki Institute of Life Science (HiLIFE), University of Helsinki, Helsinki 00014, Finland; ‡ Center for Life Sciences, National Laboratory Astana, Nazarbayev University, Astana 010000, Kazakhstan; § Department of Microbiology, University of Helsinki, Helsinki 00014, Finland; ∥ Research Center for Translational Medicine, Sirius University of Science and Technology, Sochi 354340, Russian Federation; ⊥ Department of Biological Chemistry, Institute of Biodesign and Modeling of Complex Systems, Sechenov First Moscow State Medical University, Moscow 119991, Russian Federation; # HSE University, Faculty of Biology and Biotechnology, Moscow 117418, Russian Federation; ¶ Faculty of Bioengineering and Bioinformatics, Lomonosov Moscow State University, Moscow 119234, Russian Federation; ∇ Belozersky Institute of Physico-Chemical Biology, Lomonosov Moscow State University, Moscow 119992, Russian Federation; ○ Institute of Translational Medicine and Biotechnology, Sechenov First Moscow State Medical University, Moscow 119991, Russian Federation

## Abstract

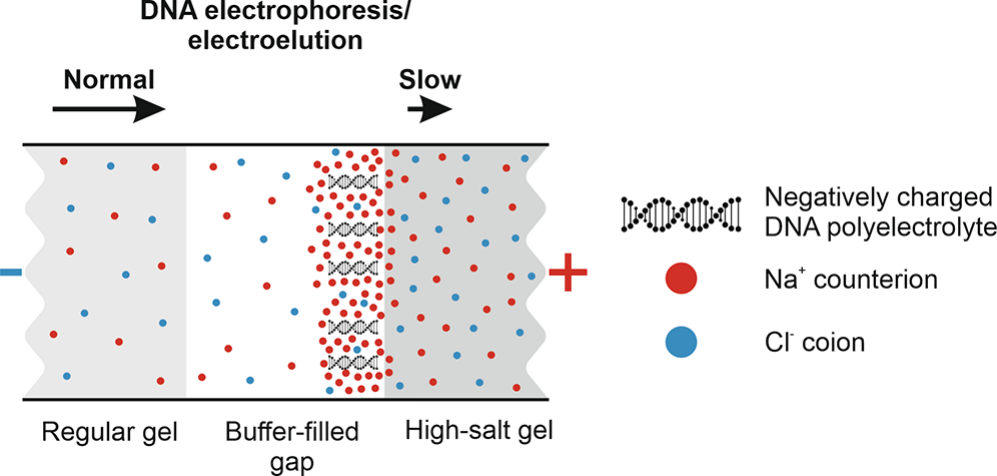

Long-read sequencing technologies require high-molecular-weight (HMW) DNA of sufficient purity and integrity, which can be difficult to obtain from complex biological samples. We propose a method for purifying HMW DNA that takes advantage of the fact that DNA’s electrophoretic mobility decreases in a high-ionic-strength environment. The method begins with the separation of HMW DNA from various impurities by electrophoresis in an agarose gel-filled channel. After sufficient separation, a high-salt gel block is placed ahead of the DNA band of interest, leaving a gap between the separating gel and the high-salt gel that serves as a reservoir for sample collection. The DNA is then electroeluted from the separating gel into the reservoir, where its migration slows due to electrostatic shielding of the DNA’s negative charge by excess counterions from the high-salt gel. As a result, the reservoir accumulates HMW DNA of high purity and integrity, which can be easily collected and used for long-read sequencing and other demanding applications without additional desalting. The method is simple and inexpensive, yields sequencing-grade HMW DNA even from difficult plant and soil samples, and has the potential for automation and scalability.

## Introduction

Isolation and purification of DNA from biological samples represent the first step in a wide range of molecular biology protocols used in genetics, molecular medicine, forensics, and biotechnology. The success of these protocols often depends on the initial separation of DNA from various impurities, including peptides and proteins (e.g., nucleases), oligonucleotides, polysaccharides, polyphenols, lipids, pigments, humic substances, secondary metabolites, various enzyme inhibitors, etc. One application that is highly dependent on DNA purity is nucleotide sequencing, which is arguably the most dynamically evolving area in the field of bioinstrumentation. During the eras of Sanger and short-read massive parallel sequencing, the availability of high-quality DNA was an important requirement for obtaining good sequencing results. This requirement is still applicable to modern third-generation sequencing (TGS) platforms,^1^ which can generate reads of tens to hundreds of thousands of base pairs. However, to fully benefit from such long reads, the sequenced DNA must be not only of high quality but also of sufficient length. Thus, DNA integrity is just as important as the purity for any method intended to produce DNA for long-read sequencing.

Numerous experimental protocols for DNA isolation and purification have been described in the literature.^2−5^ These protocols typically include a cell lysis step, followed by DNA separation from various impurities. The most commonly used separation strategies broadly fall into three categories: DNA precipitation, liquid phase extraction, and solid phase extraction. The first separation strategy capitalizes on the ability of polar solvents (e.g., ethanol) and salts (e.g., sodium acetate) to precipitate DNA from aqueous buffers.^6^ Although this strategy is suitable for the purification of high-molecular-weight (HMW) DNA and is even used in commercial kits, the yield and purity of HMW DNA can be suboptimal, particularly for complex samples. In the liquid phase extraction, polar DNA remains in the aqueous phase, while nonpolar impurities such as proteins and lipids are partitioned to an organic phase (usually phenol–chloroform) or the aqueous–organic interface.^7^ To obtain HMW DNA of acceptable purity, the organic extraction procedure is often repeated several times, resulting in lower yields and longer exposure to hazardous chemicals. In the solid phase extraction, DNA is bound to a solid support, while impurities are removed by washing. Although many different types of solid supports have been successfully used for DNA purification, silica has become especially popular over the past few decades. Commercial kits based on solid phase extraction are widely available, and their advantages include reduced sample processing time and parallel processing of a large number of samples. However, they still require multiple steps, proprietary chemicals, columns, and specialized equipment, such as centrifuges or vacuum filtration devices. Conventional solid phase extraction protocols and commercial kits are generally not suitable for the isolation of HMW DNA. Modified protocols have been developed specifically for this purpose, but they are typically more complicated and the corresponding commercial kits are more expensive. Thus, a number of in-house and commercial methods are available for HMW DNA isolation and purification, but all of them have limitations: they are often time-consuming and involve multiple steps, can be too expensive for budget-conscious laboratories, some of them require the use of hazardous chemicals, and may not produce HMW DNA of sufficient quality and yield from complex biological samples.

One approach to obtaining high-quality DNA from complex samples is gel electrophoresis, followed by electroelution from excised gel pieces. The most common variant of this method is electroelution into dialysis bags.^8,9^ However, the substantial labor and time constraints associated with this approach limit its use in everyday laboratory practice. Another strategy for obtaining DNA of high quality from complex samples employs electrophoresis, followed seamlessly by electroelution. One example is the SageELF system (Sage Science Inc., USA), in which DNA is loaded onto a precast agarose gel cassette, and following electrophoresis, the separated DNA fragments are electroeluted from the gel using the laterally positioned electrodes into 12 individual sample collection wells. The resulting DNA fractions can be used for nucleotide sequencing and other demanding applications without additional purification. Although SageELF is a powerful tool for DNA fractionation and purification, its drawbacks include costly equipment and supplies, an upper molecular-weight cutoff of 40 kb, and an inability to simultaneously process a large number of samples.

The method we propose here also involves agarose gel electrophoresis, seamlessly followed by electroelution. It is based on the fact that the electrophoretic mobility of DNA decreases with increasing salt concentration in the electrophoresis medium. Following sufficient electrophoretic purification of DNA in a separating gel, a block of high-salt gel is placed ahead of the DNA in its electrophoretic path, leaving a gap between the separating gel and the high-salt gel. The current is reapplied until the target DNA migrates into the gap, which we refer to as the sample collection reservoir, where it slows and accumulates. The purified DNA can be easily collected from the reservoir by pipetting and used either immediately or after desalting, depending on the downstream application. The proposed DNA purification method is particularly useful when other methods are ineffective or impractical, e.g., for processing difficult samples containing complex mixtures of chemically diverse biomolecules. Moreover, the method is cost-effective and does not require sophisticated reagents or instrumentation, mostly utilizing existing gel electrophoresis equipment.

## Experimental Section

### HMW DNA Purification Protocol

A 2 L bottle of 1× THE running buffer was prepared by adding 40 mL of 50× THE buffer to 1960 mL of ultrapure water.

1. To cast the 0.8% separating gel, 0.8 g of ultrapure agarose was added to 100 mL of 1× THE buffer in an Erlenmeyer flask. The flask was swirled gently for mixing. The volume of the solution, designated further as agarose solution 1 (AS1), was more than sufficient for casting five gel channels. The flask was heated with AS1 in a microwave oven at 800 W with occasional gentle swirling until all the agarose dissolved, forming a clear solution. The molten agarose was kept at 55–60 °C for further use.
2. To cast the 0.8% high-salt gel, 0.8 g of ultrapure agarose was added to 90 mL of 1× THE buffer in an Erlenmeyer flask. The flask was swirled gently for mixing. The solution was further designated as agarose solution 2 (AS2). The flask was heated with AS2 in a microwave oven at 800 W with occasional gentle swirling until all the agarose dissolved, forming a clear solution. 10 mL of 5 M NaCl was added to the solution and the molten agarose kept at 55–60 °C for further use.
3. A single-well electrophoresis comb was inserted into the top of the gel molding tray. The solution AS1 was poured into the tray, filling the entire mold cavity and taking care not to trap any air bubbles below the comb’s tooth. The gel was allowed to solidify at room temperature, forming a 1 × 1 cm wide horizontal agarose separation channel.
4. A 2 cm long section of the gel was excised at a distance of ∼4 mm downstream of the comb with a clean scalpel, creating a sample collection reservoir. Another 1 cm long section of the gel was excised ∼3 mm further downstream of the collection reservoir, creating a mold for the high-salt gel. The solution AS2 was poured into the mold cavity followed by waiting for the gel to solidify at room temperature.
5. The “sandwich” of the high-salt gel and two flanking blocks of the low-salt gel were carefully removed with a spatula, and the low-salt gel spacer was cut off with a scalpel and discarded. The high-salt gel was put aside for further use.
6. The gel tray was placed into an electrophoresis tank with the comb oriented toward the cathode. The tank was filled with 1× THE buffer until the gel was just covered. The comb was carefully removed by pulling it straight up. Flooding the gel with buffer prior to removing the comb prevented the walls of the loading well from deforming and sticking to each other.
7. The crude DNA sample (e.g., SDS/proteinase K- or CTAB-extracted) was mixed with 10× loading buffer [20% (w/v) Ficoll 400, 100 mM Tris–HCl (pH 8.0), 5 mM EDTA, 0.01% xylene cyanol FF] to a final concentration of 1.5×.
8. The sample (50 μL) was carefully loaded into the well, the electrophoresis chamber lid was closed, and the electrodes were connected to a power supply.
9. The gel was run at 100 V until the tracking dye (xylene cyanol FF) had just run out of the gel. Alternatively, DNA migration was monitored during electrophoresis, and the run was stopped when the DNA of interest had reached the end of the gel. It was ensured that the DNA did not run out of the gel.
10. After the power supply had been switched off, the electrodes were unplugged, the chamber lid was opened, and the gel tray was removed from the electrophoresis tank. The remaining running buffer was discarded and the electrophoresis tank was rinsed with deionized water.
11. The excess running buffer was drained from the gel tray with a corner of a paper towel. The high-salt gel block was carefully returned to its original position in the tray.
12. The gel tray was placed back into the electrophoresis tank. The tank was filled with fresh 1× THE buffer so that the buffer level was just below the gel surface.
13. Electrophoresis was continued at 100 V for additional 20 min to elute the gel-purified DNA into the sample collection reservoir.
14. The power supply was turned off, the electrodes were unplugged, and the chamber lid was removed. The eluted DNA was collected from the collection reservoir with a pipet.

## Results and Discussion

### Theory and Proof of Concept

**Figure 1:**
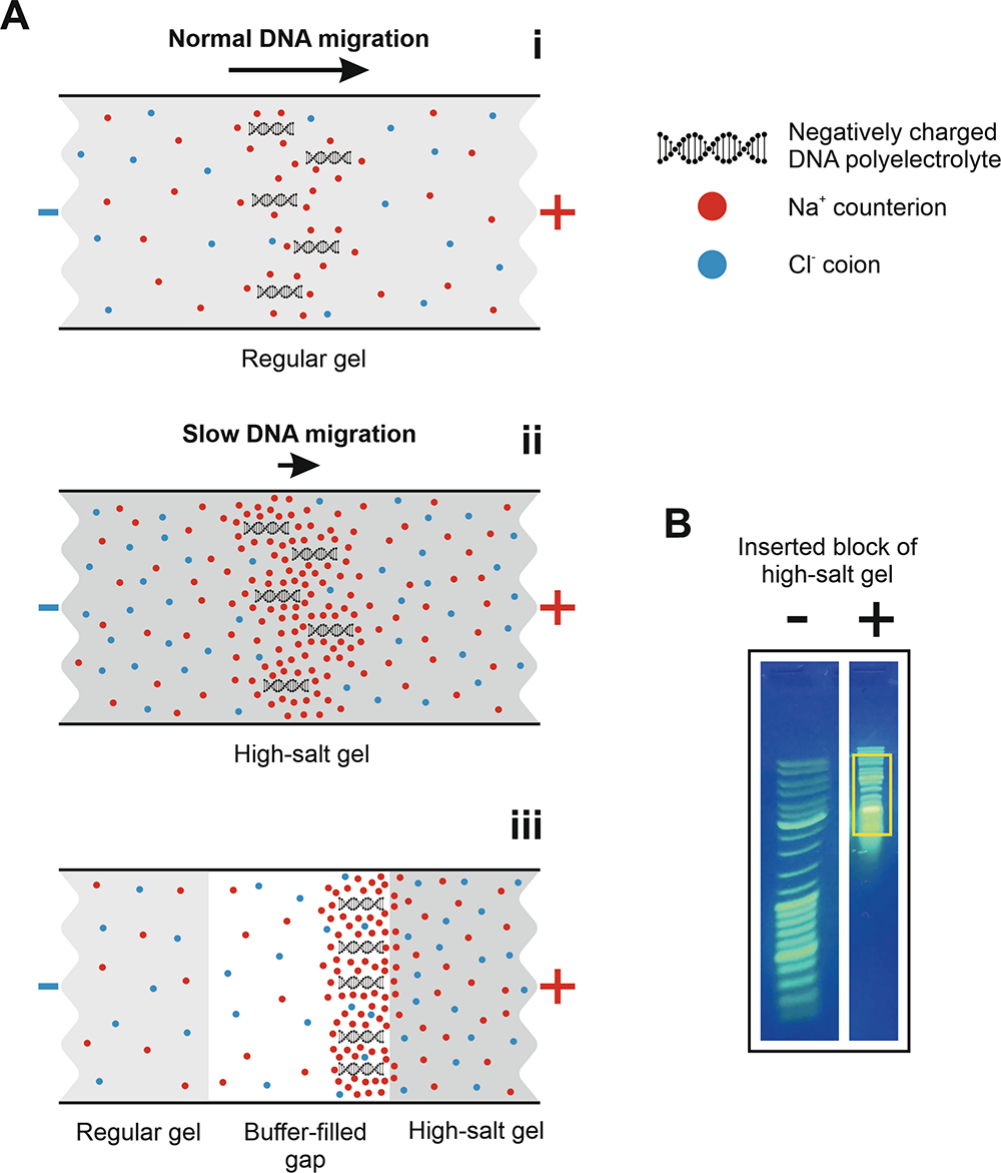
Trapping electroeluted DNA using gels of high ionic strength: theory and proof of concept. (A) Schematic representation of the ion atmosphere surrounding DNA in gels containing different salt concentrations. The red and blue circles represent Na^+^-counterions and Cl^–^-co-ions. The increased counterion accumulation around DNA in the high-salt gel (ii) compared to the regular gel (i) results in stronger electrostatic shielding of DNA’s negative charge, reducing its electrophoretic mobility. This phenomenon can be used to trap DNA in a buffer-filled gap in front of the high-salt gel (iii). (B) To demonstrate how the high-salt gel traps DNA, a gel slice (boxed in yellow) was excised upstream of the migrating DNA ladder and replaced with a gel containing 1 M NaCl. Note the decrease in electrophoretic mobility of the DNA ladder in the right lane compared to the control left lane.

In ionic solutions, highly negatively charged DNA is surrounded by positively charged counterions (cations) and negatively charged co-ions (anions), forming the so-called ion atmosphere.^10^ The ion atmosphere has a higher concentration of attracted cations (counterion accumulation) and a lower concentration of repulsed anions (co-ion depletion) and is influenced by the ionic strength of the solution. As ionic strength increases, counterions electrostatically shield DNA’s large negative charge (Figure 1A), decreasing its free solution mobility during capillary electrophoresis.^11,12^ This phenomenon has been exploited in several electroelution devices, such as the AP-eluter,^13^ the device of Zassenhaus et al.,^14^ and the now-discontinued unidirectional analytical electroeluter (International Biotechnologies, Inc. USA); in all cases, an aqueous solution of high ionic strength served as a DNA trap. A more recent example of this methodology is the protocol of Zarzosa-Alvarez et al.,^15^ in which an agarose gel slice containing the DNA fragment of interest is placed into an electroeluter and its V-shaped channel is filled with a high-salt buffer. During electroelution, the DNA fragment migrates from the agarose slice into the salt trap. At the final stage of the protocol, the high-salt buffer containing electroeluted DNA is collected with a pipet and precipitated with ethanol. Although this protocol gives an acceptable sample quality and yield, it requires specialized equipment (an electroeluter) and is relatively labor-intensive and time-consuming.

Based on the principle behind the above methodology, we hypothesized that placing a block of high-salt gel in front of a migrating DNA band would reduce its electrophoretic mobility. To test this hypothesis, we excised a gel slice upstream of the migrating DNA and replaced it with a gel containing a high concentration of salt. When DNA reached the high-salt gel, its electrophoretic mobility decreased significantly, indicating electrostatic shielding by excess counterions (Figure 1B). Based on this proof of concept, we developed a method for HMW DNA purification that combines gel electrophoresis and electroelution and involves DNA trapping against a high-salt gel barrier.

### Method Implementation

**Figure 2:**
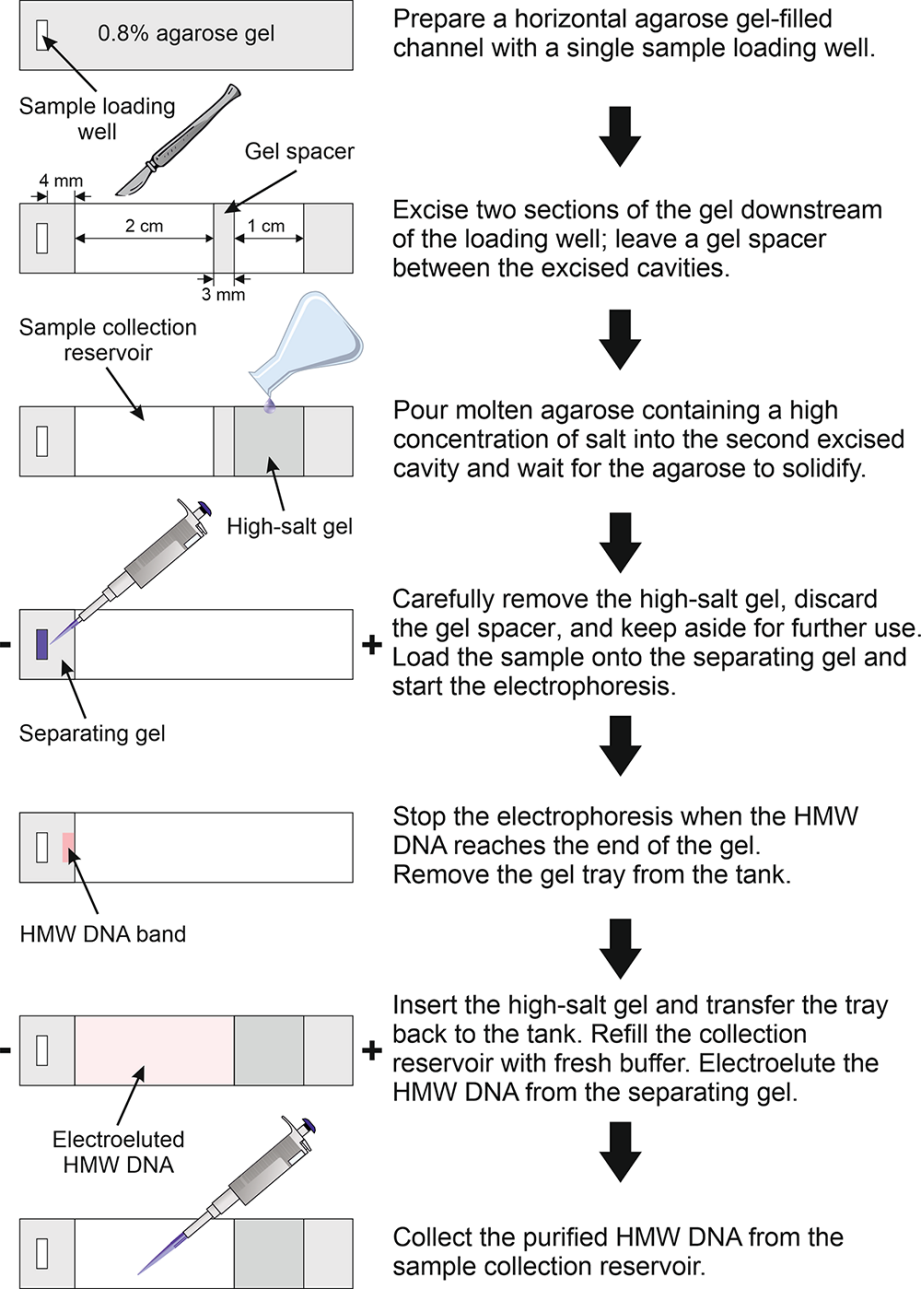
Schematic representation of the proposed HMW DNA purification workflow.

The proposed method employs HMW DNA purification by electrophoresis in horizontal agarose gel-filled channels. A molding tray for these channels can be conveniently fabricated in-house by using 3D printing. Each channel is approximately 1 cm wide, allowing for the use of a single-well electrophoresis comb. Filling the mold cavities with molten 0.8% agarose in running buffer (see below for buffer details) generates a row of parallel open-top gel channels, each with one sample loading well. For the sake of clarity, we hereafter describe the purification of HMW DNA in a single gel channel. Two sections of the gel are excised downstream of the loading well, creating a sample collection reservoir and a mold cavity for the high-salt gel (Figure 2). A thin (∼3 mm) gel spacer between the two excised sections serves as a liquid-proof seal for the mold cavity. The mold cavity is then filled with a molten agarose solution containing a high concentration of salt. After the gel has solidified, the gel “sandwich” is carefully removed from the molding tray, the gel spacer is discarded, and the high-salt gel is stored for later use during electroelution. The tray, which now contains only the separating gel, is transferred to an electrophoresis tank and covered with running buffer. A crude sample containing the HMW DNA of interest (e.g., the SDS/proteinase K-extracted sample or the CTAB-extracted soil/wood sample) is loaded into the loading well and subjected to electrophoresis. When the HMW DNA reaches the end of the separating gel, the current is temporarily turned off and the tray is removed from the electrophoresis tank. The high-salt gel block is inserted back into its original position (Figure 2), and the tray is returned to the electrophoresis tank. The sample collection reservoir is refilled with fresh running buffer, and the current is turned back on to elute the purified HMW DNA from the separating gel into the reservoir. After entering the buffer-filled reservoir, DNA migration gradually slows due to the neutralization of its charge by excess counterions from the high-salt gel. This causes the gel-purified HMW DNA to accumulate in the reservoir, from which it can be easily collected by pipetting.

### DNA Yield and Purity

Isolating HMW DNA from complex biological samples is often time-consuming and labor-intensive and results in low yield and/or purity. It is especially difficult to isolate HMW DNA from plant and soil samples. Plant cells contain polysaccharides and polyphenols that are hard to separate from DNA, making HMW DNA extraction from plants much more difficult than extraction from animals. However, HMW DNA extraction from soil presents an even greater challenge due to the presence of fragmented DNA and coextracting humic substances. Despite these challenges, the proposed method has proven to be capable of producing HMW DNA with a high yield and purity even from complex plant and soil samples.

**Figure 3:**
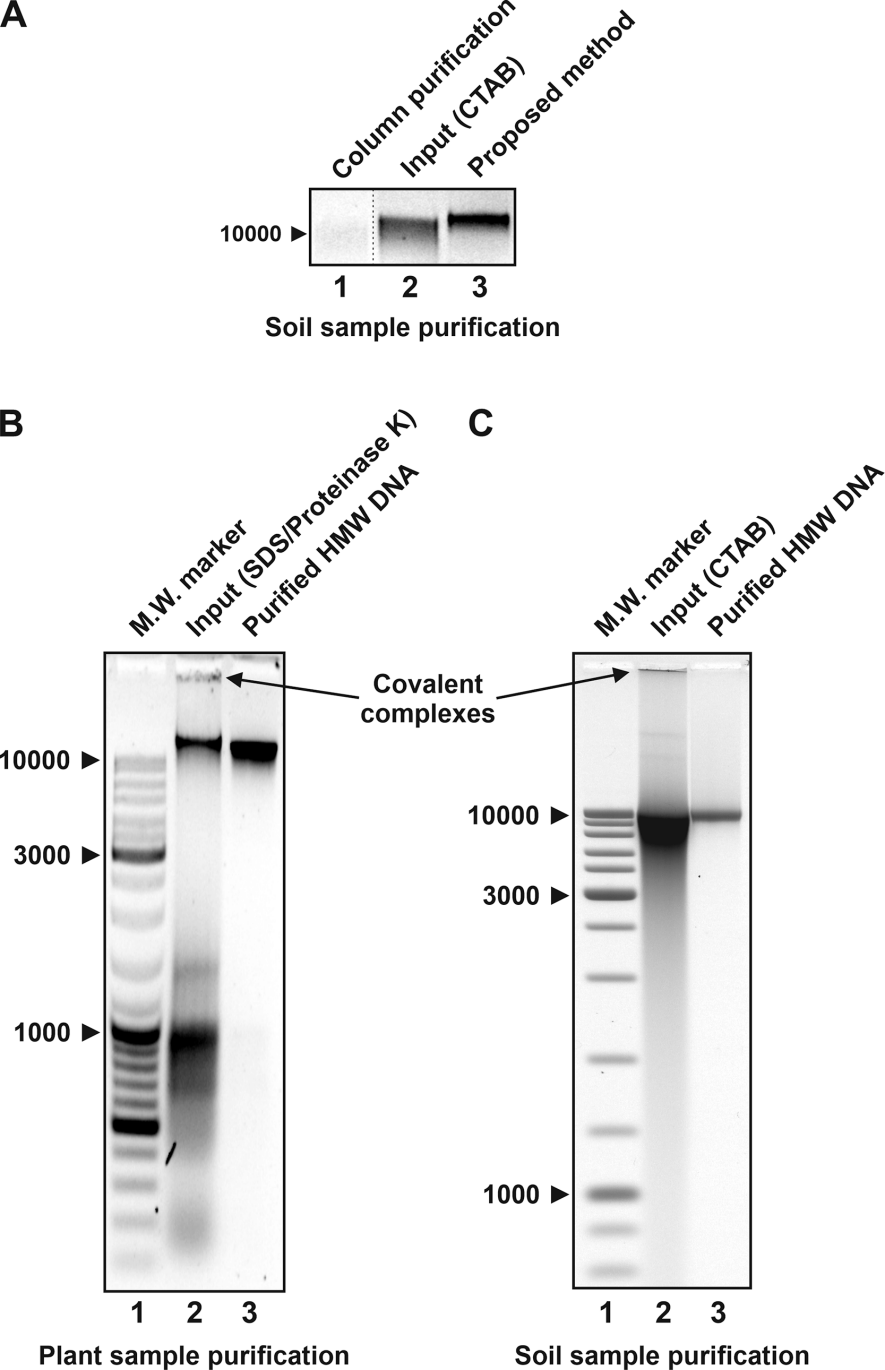
Yield and purity of HMW DNA obtained from difficult samples using the method proposed in this study. (A) The proposed method extracts more HMW DNA from a complex soil sample than a commercial column purification kit. Shown is a negative image of an ethidium bromide-stained agarose gel. Lane 1: 1/10 aliquot of ∼90 ng of HMW DNA isolated using the E.Z.N.A. soil DNA extraction kit from a soil sample containing ∼1.5 μg of total DNA (HMW DNA yield around 6%). Lane 2: ∼100 ng of CTAB-extracted DNA from the same soil sample. ∼10 μg of this crude DNA preparation was used as input for HMW DNA purification using the proposed method. Lane 3: 1/100 aliquot of ∼3 μg of HMW DNA isolated using the proposed method from ∼10 μg of the CTAB-extracted DNA (HMW DNA yield around 30%). (B) The proposed method yields high-purity HMW DNA from a complex plant sample, as determined by agarose gel electrophoresis. Lane 1: molecular weight marker (GeneRuler DNA ladder, Thermo Fisher Scientific). Lane 2: crude nucleic acid preparation extracted with SDS/proteinase K from *Zingeria trichopoda* leaves, which served as an input for HMW DNA purification using the proposed method. Lane 3: purified HMW DNA. Note the absence of low-molecular-weight nucleic acids and heavy covalent complexes in the purified sample. (C) Same as (B) except that crude, CTAB-extracted DNA from a complex soil sample was used as an input for HMW DNA purification. Note the absence of a continuous smear of fragmented DNA as well as heavy covalent complexes in the purified sample (lane 3). Molecular weight marker sizes are indicated in base pairs to the left of each panel. The black line in lane 2 of (B) is caused by a dust particle captured by the UV camera and has no effect on data interpretation.

Most current methods for purifying HMW DNA from plant or soil samples yield less than 10% of the total starting DNA. In the case of column purification, this is because a majority of HMW DNA passes through the column without being retained. In organic extraction, HMW DNA complexes with polysaccharides and other biological macromolecules may partition to the organic phase or the aqueous–organic interface, resulting in a lower yield. Because the proposed method is based on a different principle than the above approaches, it is not constrained by these limitations and provides significantly higher HMW DNA yields, approaching 50% for plant samples and about 30% for soil samples. The fact that the proposed method yielded significantly more HMW DNA from soil than a popular column purification method (the E.Z.N.A. soil DNA Extraction Kit, Omega Biotek, Inc., Norcross, GA, USA) demonstrates the method’s ability to efficiently process even the most difficult samples (Figure 3A). We repeatedly obtained yields close to or even exceeding 30% from a variety of soil samples, confirming the efficacy and reproducibility. Finally, it should be noted that gel overloading is the primary cause of DNA loss in the proposed method, resulting in band smearing and incomplete recovery of the trailing DNA. This problem, however, can be easily avoided by controlling the amount of DNA loaded on the gel.

Gel electrophoresis represents the method of choice for the purification of long oligonucleotides because it offers one of the highest levels of purity among the available purification methods. The same is generally true for the purification of full-length DNA, unless the method involves gel grinding (e.g., the “crush and soak” technique). Mechanical grinding of an agarose gel can contaminate DNA with gel particles and soluble polysaccharide sulfates, which may inhibit subsequent enzymatic reactions.^16,17^ The method proposed in this study does not involve gel grinding; instead, it relies on a continuous process of electrophoresis and electroelution that does not disturb the gel matrix, thereby reducing concerns over polysaccharide contamination. Indeed, the *A*_260_/*A*_230_ ratio for HMW DNA isolated from complex soil samples using the proposed method was around 2.0, indicating that the DNA was essentially free from polysaccharides. In contrast, using a commercial column purification method (the E.Z.N.A. soil DNA Extraction Kit) to purify the same soil samples yielded HMW DNA with *A*_260_/*A*_230_ ratios of less than 1, indicating the presence of organic contaminants such as humic acids, polyphenols, polysaccharides, pigments, peptides, etc. In addition to spectrophotometry, we assessed the quality of the obtained HMW DNA by agarose gel electrophoresis. The electrophoretic analysis confirmed that the proposed method produced HMW DNA with high purity and integrity. As shown in Figures 3 and S1, HMW DNA isolated from complex plant samples was essentially free of low-molecular-weight nucleic acids. Furthermore, our method successfully separated full-length DNA from its degradation fragments, which are frequently present in soil samples and are difficult to remove using other purification techniques (Figures 3C and S1). Finally, gel electrophoresis showed that the proposed method could separate HMW DNA from its covalent complexes with other biological macromolecules, such as oxidized polyphenols (Figures 3B,C and S1). Because of their covalent nature, these complexes are useless for molecular studies,^18^ but traditional HMW DNA purification methods will struggle to remove them from DNA preparations. To summarize, the proposed method is capable of producing HMW DNA with high yield, integrity, and purity, making it suitable for a variety of downstream assays such as nucleotide sequencing (Table S1), PCR, ligation, restriction digestion, etc.

### Running Buffer

In the proposed method, the electroeluted DNA accumulates in the sample collection reservoir filled with the electrophoresis running buffer. Therefore, the choice of a running buffer is important if the DNA is to be used in downstream applications without additional purification. The buffer must be compatible with subsequent enzymatic reactions as well as with long-term sample storage. Tris-borate (TBE) is one of the most commonly used running buffers used in nucleic acid electrophoresis. However, borate ions form complexes with DNA^19^ and proteins^20^ and interfere with the activity of many enzymes.^21^ This makes TBE unsuitable for our protocol. The most popular alternative to TBE is the Tris-acetate (TAE) buffer. However, TAE contains 20 mM acetate, which may inhibit thermostable DNA polymerases^22^ and a number of DNA-modifying enzymes such as alkaline phosphatase^23^ and S1 nuclease.^24^ Another disadvantage of TAE is its low buffering capacity,^25^ which makes it difficult to run gels for extended periods of time. Prolonged electrophoresis in a narrow channel gradually depletes the TAE buffering capacity, causing agarose degradation and polysaccharide release into the collection reservoir as the pH rises. For the reasons stated above, we decided against using TAE in favor of the Tris-HEPES (THE) buffer [20 mM Tris, 20 mM HEPES, and 0.1 mM EDTA (optional), pH 8.0], which has a higher buffering capacity and keeps the pH more stable during extended electrophoresis in a narrow channel. Furthermore, because THE buffer is less prone to enzyme compatibility issues than TBE or TAE, DNA dissolved in it can be directly used in various enzymatic reactions. Indeed, we were able to successfully use HMW DNA electroeluted in THE buffer for nucleotide sequencing as well as other enzymatic reactions, such as PCR and cloning.

### Choosing When to Insert the High-Salt Gel

Knowing when to insert the high-salt gel trap is critical to the method’s success because the trap is permanently inserted at a fixed distance downstream of the separating gel, and its position after that remains constant (Figure 2). The trap should be inserted once all of the smaller-sized impurities, such as RNA, oligonucleotides, and other biological macromolecules, have been eluted from the separating gel, and the target HMW DNA is nearing the end of the gel. The easiest and most straightforward way to roughly estimate when the HMW DNA is going to approach the end of the separating gel is by using a slower-migrating tracking dye such as xylene cyanol. Since xylene cyanol migrates in a 0.8% agarose gel at about the same rate as a 5 kb dsDNA fragment, the high-salt gel should be inserted once xylene cyanol begins to run off the separating gel. Alternatively, it is possible to determine when the HMW DNA reaches the end of the separating gel by ethidium bromide (EtBr) staining. The obtained parameters (gel running time and voltage) could then be used to determine when to insert the high-salt gel during the isolation of similar but unstained DNA. This approach can be especially valuable for the parallel isolation of similar-sized HMW DNA in large-scale experiments. However, in some cases, such as when individual HMW DNA molecules are separated, the accuracy of DNA electrophoretic mobility estimates may be insufficient, necessitating a more precise method of locating the DNA band of interest. The most common method for detecting DNA in a gel is staining with ethidium bromide, which is still the most widely used fluorescent intercalating dye in DNA electrophoresis despite being a known carcinogen and requiring gel exposure to UV light, which causes DNA damage. Alternatives to EtBr include, among others, SYBR Green, SYBR Gold, SYBR Safe, and Eva Green, all of which have better safety profiles than that of EtBr and can be visualized with green/blue light. Nevertheless, due to their DNA-binding nature, all intercalating dyes alter the structure and mechanical properties of DNA^26^ and can potentially interfere with subsequent enzymatic applications.^27^ Therefore, if the downstream application is sensitive to the presence of an intercalating dye, we recommend removing it with an additional purification step (see below).

### Salt Concentration in the Trapping Gel

When the concentration of salt in the trapping gel is selected, a delicate balance must be struck. On the one hand, the salt concentration should be sufficient to slow down the migration of DNA in the sample collection reservoir. Too much salt, on the other hand, will contaminate the sample and may cause DNA to stall in the separating gel before it reaches the collection reservoir. After performing a series of tests, we determined that the optimal salt concentration for a gel volume of 1 cm^3^ is between 0.5 and 1 M sodium chloride. This range agrees with previously published findings that the free-solution electrophoretic mobility of dsDNA decreases with increasing ionic strength until it begins to level off at about 0.6 M sodium acetate.^12^ At 0.5 M sodium chloride in the gel, the concentration of sodium ions in the recovered DNA sample is about 30 to 40 mM, as determined by inductively coupled plasma optical emission spectroscopy. Even salt-sensitive enzymatic reactions, such as ligation with T4 DNA ligase, are compatible with this concentration of salt, especially when the sample dilution in the reaction mixture is taken into account. Therefore, sample desalting is not necessary for most downstream enzymatic applications, including adaptor ligation for long-read sequencing. Only a few applications that are particularly sensitive to salt may require an additional sample desalting step.

Ethanol precipitation is considered the first method of choice for DNA desalting and concentration. It does not require expensive equipment or reagents but is relatively time-consuming and may result in incomplete DNA recovery from dilute samples. One may therefore consider alternative desalting techniques such as gel filtration on a spin column followed, if necessary, by concentrating the sample using vacuum drying. Another approach suitable for simultaneous sample desalting and concentration is the use of commercial centrifugal filter devices (e.g., a Millipore Amicon Ultra-2 centrifugal filter unit). Thus, several methods are available for efficient HMW DNA desalting and concentration. Notably, these methods will also remove any residual tracking or intercalating dyes from the HMW DNA of interest.

### Advantages and Potential Applications of the Method

The main advantage of the proposed method is that it offers a simple and efficient solution for purifying HMW DNA for long-read sequencing from even the most difficult samples. The current long-read/TGS technologies were developed to address the major limitation of second-generation sequencing, namely, the generation of short reads less than 600 nucleotides. The longer reads (ranging from 10 kb to more than 4 Mb) generated by TGS substantially improve the quality and completeness of the genome assembly and are particularly useful for the characterization of highly repetitive genomic regions. However, the current TGS chemistry is sensitive to the presence of impurities in the DNA samples. Therefore, it is important to obtain DNA that is both highly pure and of high molecular weight to take advantage of the opportunities offered by TGS. The success of a TGS run depends on the integrity or degree of fragmentation of the DNA molecules used for library preparation. Because shorter DNA fragments compete with longer ones for pore occupancy in the flow cell, any HMW DNA extraction method intended for long-read sequencing should attempt to maximize the share of long and ultralong DNA molecules. Our protocol has the advantage of effectively removing short DNA molecules (less than 10 kb) prior to sequencing, thereby increasing the fraction of long reads. This is especially important for sequencing difficult samples containing fragmented DNA, such as soil, feces, ancient wood, etc.

Although the 0.8% agarose gels used in this study have an upper resolution limit of about 15 kb, larger DNA molecules can still be recovered from such gels, provided that the DNA enters the gel and migrates without being sieved in a process called reptation.^28^ Thus, the proposed method can successfully separate DNA molecules larger than 15 kb from smaller impurities. Indeed, by removing such impurities, we were able to obtain DNA of high purity with a length of >50 kb from complex plant and soil samples. However, if individual DNA molecules larger than 15 kb must be separated from one another, then the upper resolution limit of gel electrophoresis must be increased. In theory, this can be accomplished by using low percentage agarose gels (0.1–0.3%), which can resolve DNA molecules up to several Mbp in length.^29^ However, such gels are fragile, require special handling, and take a long time to run. Another method for increasing the upper resolution limit of agarose gels is pulsed-field gel electrophoresis (PFGE), which involves the application of a periodically alternating current from different directions. In contrast to conventional steady-field electrophoresis, PFGE can separate HMW DNA up to 5 Mb in length.^30^ Although not tested here, we believe that our method can be used to isolate HMW DNA separated by PFGE. While there may be practical challenges, there are no theoretical obstacles to using the high-salt gel electroelution trap in conjunction with PFGE. Thus, the method proposed in this study has the potential to resolve and isolate HMW DNA up to several megabases in size with a purity suitable for TGS and other demanding downstream applications.

Another advantage of the proposed method is its ability to separate the HMW DNA of interest from chemically diverse biomolecules in a single step. These molecules include, among others, unwanted DNA and RNA, short DNA and RNA oligonucleotides, polysaccharides, polyphenols, humic substances, proteins, peptides, lipids, pigments, and secondary metabolites. Conventional DNA purification methods often require multiple steps when dealing with complex samples. In contrast to these methods, gel electrophoresis can separate target DNA from a variety of chemically related and unrelated molecules in a single step, based on differences in charge and size. In addition, gel electrophoresis does not usually require extensive preliminary sample processing to achieve a good separation. In our hands, straightforward and inexpensive SDS/proteinase K or CTAB DNA extraction methods were adequate for preparing crude samples for gel loading. Notably, proteinase K and ionic detergents inactivate DNases in cell lysates, increasing the DNA yield and facilitating the recovery of long, undegraded DNA required for long-read sequencing. Another advantage of DNA purification by gel electrophoresis is that it requires less starting DNA than the other purification methods. This is especially useful when the purified sample contains small quantities of target DNA. Furthermore, as previously discussed, loading more than 10 μg of DNA on a gel will result in band smearing, a problem that will worsen as the DNA size increases. Therefore, we recommend using less starting material than is required by other methods. In light of the above, the proposed method holds promise for single-step processing of difficult samples containing chemically diverse impurities and only small quantities of target DNA.

**Figure 4:**
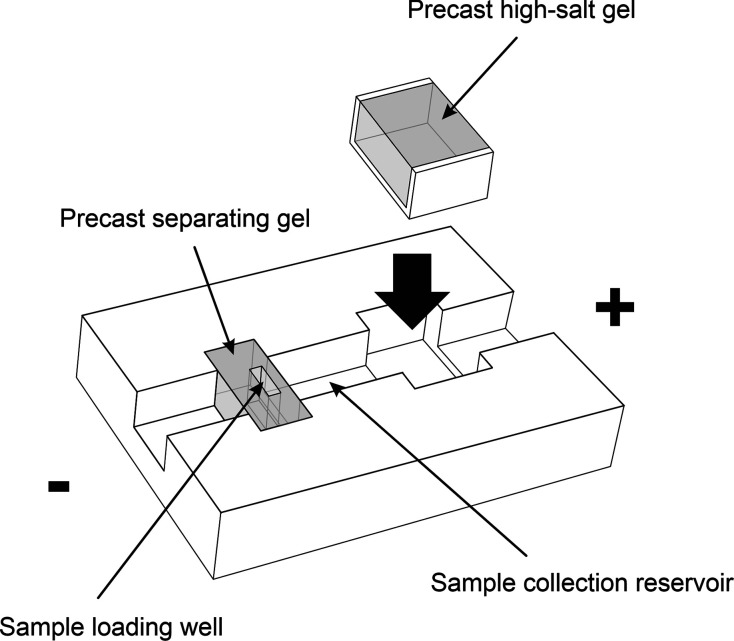
Model of an electrophoresis/electroelution device for large-scale HMW DNA purification. The device has a channel with two troughs, the first of which contains a precast separating agarose gel. A complex sample containing the HMW DNA of interest is loaded on the gel. Electrophoresis is allowed to proceed until all undesired molecules have migrated out and the HMW DNA has reached the end of the gel. The electrophoresis is paused, the sample collection reservoir is replenished with fresh buffer, and a tray containing a high-salt gel is inserted into the second trough downstream of the separating gel. The current is reapplied until the HMW DNA migrates out of the separating gel and into the buffer-filled collection reservoir, where it slows down and accumulates. The device can be scaled up to accommodate multiple separation channels. Much of the workflow, including sample loading, buffer refilling, high-salt gel insertion, and DNA recovery from the collection reservoir, can be automated.

An important feature of the proposed method is its potential for scalability and automation. In this study, we used the horizontal electrophoresis system with five agarose gel-filled separation channels. However, it should be possible to scale up the system to accommodate 12, 24, 48, or even more separation channels and optimize the channel molds for specific high-throughput tasks. Following initial optimization with 3D printing, a suitable prototype can be computer numerical control (CNC)-machined from a UV-transparent plexiglass. Figure 4 shows a descriptive model of the separation channel designed for large-scale purification of HMW DNA. The channel has two troughs, the first of which is prefilled with an agarose gel. This gel contains a sample loading well and serves as the electrophoresis medium to separate HMW DNA from impurities. The second trough is intended for the insertion of a plastic tray with a precast high-salt gel. When HMW DNA reaches the end of the separating gel, the run is paused, and the tray containing the high-salt gel is inserted into the second trough. This procedure is easy to automate, unlike the insertion of a gel directly into the separation channel. The current is then reapplied until the HMW DNA migrates out of the separating gel and into the buffer-filled collection reservoir (Figure 4), where it slows down and accumulates. Once again, the task of collecting DNA from the reservoir can be easily automated through robotics.

## Conclusions

The method proposed in this study can become a useful tool to isolate HMW DNA for long-read sequencing and other demanding applications from eukaryotic and prokaryotic cells, mitochondria, chloroplasts, large viruses, and other sources. It can be used when other methods are impractical or ineffective, such as when purifying HMW DNA in a single step from complex biological samples containing large quantities of chemically diverse impurities and only small amounts of target DNA. Moreover, the method is cost-effective and scalable and has potential for automation.

## Data Availability

The original gel electrophoresis data from this study are publicly available in Zenodo at 10.5281/zenodo.8267312, reference number [8267312]. The original ONT data reports are openly available in Figshare at 10.6084/m9.figshare.c.6798126.v1.

## Supporting Information Available

Additional methods, including in-house SDS/proteinase K- and CTAB-based DNA extraction protocols, results of gel electrophoresis analysis of HMW DNA isolated from complex soil and plant samples, and the Oxford Nanopore Technologies (ONT) sequencing report (PDF)

## Notes

The method proposed in this study is the subject of a pending patent application.
