# Supplementary material for: Isolation of High-Molecular-Weight DNA for Long-Read Sequencing Using a High-Salt Gel Electroelution Trap
Source: Anal Chem. 2023 Nov 22;95(48):17818–25. doi: 10.1021/acs.analchem.3c03894 (PMC10701706; doi:10.1021/acs.analchem.3c03894)
Supplement: Supplementary file 1 — ac3c03894_si_001.pdf [file ac3c03894_si_001.pdf]

Isolation of High Molecular Weight DNA for Long-Read Sequencing Using a High-Salt Gel Electroelution Trap

Ruslan Kalendar[1,2*], Konstantin I. Ivanov[3,4], Olga Samuilova[5,6], Ulykbek Kairov[2], Andrey A. Zamyatnin Jr.[4,7,8,9]

[1] Institute of Biotechnology, Helsinki Institute of Life Science (HiLIFE), University of Helsinki, Helsinki, Finland;

[2] Center for Life Sciences, National Laboratory Astana, Nazarbayev University, Astana, Kazakhstan

[3] Department of Microbiology, University of Helsinki, Helsinki, Finland;

[4] Research Center for Translational Medicine, Sirius University of Science and Technology, Sochi, Russian Federation;

[5] Department of Biochemistry, Sechenov First Moscow State Medical University, Moscow, Russian Federation;

[6] HSE University, Moscow, Russian Federation;

[7] Faculty of Bioengineering and Bioinformatics, Lomonosov Moscow State University, Moscow, Russian Federation;

[8] Belozersky Institute of Physico-Chemical Biology, Lomonosov Moscow State University, Moscow, Russian Federation;

[9] Institute of Translational Medicine and Biotechnology, Sechenov First Moscow State Medical University, Moscow, Russian Federation.

*Corresponding author:
Ruslan Kalendar, ruslan.kalendar@helsinki.fi, phone: +358504483505

Additional methods, including the in-house SDS/Proteinase K- and CTAB-based DNA extraction protocols, as well as the Oxford Nanopore Technologies (ONT) sequencing report.

Supplementary Methods

Control of HMW DNA Quality and Integrity

The ratio between absorbance at 260 nm and at 230 nm (A260/A230) was calculated to estimate DNA purity. The absorbance values were determined using a NanoDrop ND-2000 spectrophotometer (Thermo Fisher Scientific). HMW DNA concentrations measured using NanoDrop were validated with a Qubit 3.0 fluorometer (Invitrogen) using the dsDNA BR assay kit (Thermo Fisher Scientific). DNA integrity was assessed by electrophoresis on 1% agarose gels.

Long-Read Sequencing with Oxford Nanopore Technologies (ONT)

Sequencing libraries were prepared using the Rapid Barcoding kit (SQK-RBK110.96, Oxford Nanopore Technologies) and the solid-phase reversible immobilization (SPRI) beads for DNA clean-up following the manufacturer's instructions. Duplicate runs with different barcodes were performed for each sample, and a single sample was pooled for sequencing on a single flow cell. Each library containing ~0.5 µg of DNA was sequenced using the ONT GridION platform with R9.4.1 chemistry. Sequencing ran for 25 hours until the flow cell buffer was exhausted. Data acquisition, real-time analysis, and sample tracking were carried out using the MinKNOW (v. 2.1) software. High-accuracy base calling was performed from the fast5 files using the Oxford Nanopore Guppy tool (v. 3.0.4). The run was monitored using RAMPART (https://github.com/articnetwork/rampart), enabling it to stop the run once a minimum sequencing depth of 20x was achieved.

Sodium Content Measurements

Sodium content measurements were carried out by inductively coupled plasma optical emission spectroscopy (ICP-OES) using an Agilent 720 instrument (Agilent Technologies, Inc., Santa Clara, CA, USA). The following sodium atomic lines were selected: 568.821 nm, 588.995 nm and 589.592 nm. The average sodium content was calculated from two replicates. Prior to analysis, all samples were diluted 20-fold with ultrapure water. The accuracy was estimated using spiked sample standards following the ISO 5725-4 (2020) guidelines.

Supplementary Protocols

Preparation of 50X THE Running Buffer (1 M Tris, 1 M HEPES, 5 mM EDTA (optional), pH 8.0). Dissolve 121,14 g Tris-base, 238,3 g HEPES (free acid) in MilliQ water, add 10 ml 0.5 M EDTA (optional), and bring the final volume to 1 litter. The pH does not need to be adjusted and should be between 8.0 and 8.1.

DNA Extraction Using the SDS/Proteinase K Method

This protocol is used to extract crude DNA for gel loading from complex plant, animal, insect, fungal, and microbial samples. The following example shows how to extract DNA from a 100 µg leaf sample.

1. Grind the deep-frozen leaf tissue in liquid nitrogen with a pre-chilled mortar and pestle. Transfer the ground powder to a 1.5 ml microtube.
2. Add 500 µl of lysis buffer (2% SDS, 10 mM EDTA, 50 mM Tris-HCl, pH 8.0) and proteinase K to a final concentration of 100-200 µg/ml. Mix and incubate at 55°C for at least two hours.
3. Transfer a 50 µl aliquot of the lysate to a new 1.5 ml microtube and mix with loading buffer as described in step 7 of the gel purification protocol below. Freeze the remaining lysate until further use.

DNA Extraction Using the CTAB Method

This protocol is used to extract crude DNA for gel loading from complex samples (e.g., soil, feces, wood) containing a high concentration of organic compounds such as humic substances, pigments, etc. The example below shows how to extract DNA from samples weighing 1 to 5 grams, but the protocol can be scaled down accordingly.

1. Grind the deep-frozen sample in liquid nitrogen with a pre-chilled mortar and pestle. Transfer the ground powder to a 50-ml Falcon tube containing 40 ml of cetyl trimethylammonium bromide (CTAB) solution (2% CTAB, 1.5 M NaCl, 10 mM Na_3EDTA, 0.1 M HEPES-acid, pH 5.3). Thoroughly vortex and incubate overnight at 65°C.
2. Centrifuge the homogenate at 14,000 x g for 10 minutes at +4°C. While the centrifugation is in progress, prepare two new 50-ml Falcon tubes containing 20 ml of 100% isopropanol chilled to -20°C.
3. Divide the clarified supernatant into two equal 20-ml portions and transfer them to the Falcon tubes containing isopropanol. Vortex thoroughly.
4. Centrifuge at 14,000 x g for 10 minutes at +4°C.

5. Discard the supernatant without disturbing the DNA pellet.

6. Wash the pellet in each tube by adding 10 ml of 70% ethanol. Vortex briefly.

7. Centrifuge at 14,000 x g for 3 minutes at +4°C.

8. Completely remove the supernatant, but do not dry the pellet. Add 1 ml of 1 x TE (10 mM Tris-HCl, 1 mM Na_2EDTA, pH 8.0) to the pellet, vortex, and incubate at 65°C with occasional mixing until the pellet is completely dissolved. Pool the DNA solution from both Falcon tubes into one 2-ml microtube.

9. Transfer a 50 µl aliquot of the DNA solution to a new 1.5 ml microtube and mix with loading buffer as described in step 7 of the gel purification protocol below. Freeze the remaining DNA until further use.

ONT Sequencing Protocol for GridION Instrument

DNA concentration before starting was 17,0 ng/l (as measured by Qubit).

1. From each sample, take 10 µl.
2. Add 5 µl of Rapid Barcode Plate for each.
3. Incubate at 30°C for 2 min, then 80°C for 2 min
4. Transfer all samples to a 1,5 ml Eppendorf DNA LoBind tube.
5. Resuspend SPRI beads.
6. Add an equal volume of resuspended SPRI.
7. Incubate on a Hula mixer (rotator mixer) for 10 min.
8. Prepare 1ml of fresh 80% ethanol in nuclease-free water.
9. Spin down the sample and pellet on a magnet. Keep the tube on the magnet, and pipette off the supernatant.
10. Keep the tube on the magnet and wash the beads with 1ml of freshly prepared 80% ethanol without disturbing the pellet. Remove the ethanol using a pipette and discard.
11. Repeat previous step.
12. Briefly spin down and place the tube back on the magnet. Pipette off any residual ethanol. Allow drying for 30 seconds, but do not dry pellet to the point of cracking.
13. Remove the tube from the magnetic rack and resuspend the pellet by pipetting in 15 µl Elution Buffer (EB). Incubate for 10 minutes at a room temperature.
14. Pellet the beads on a magnet until the eluate is clear and colorless.
15. Remove and retain 15 µl of eluate containing the DNA library into a clean 1,5 Eppendorf DNA LoBind tube Quantify DNA concentration by using Qubit ds HS Assay kit.
16. DNA concentration after pooling and clean-up: 77,6 ng/ µl (as measured by Qubit).

17. Add 1 µl of Rapid Adapter F (RAP F).
18. Incubate at room temperature for 5 min.

Pores 1529

Position x1

Time 25 hours

Total loading mix- 94 µl (SBII-47 µl; LBII- 32 µl; Library- 15 µl)

Supplementary Figures

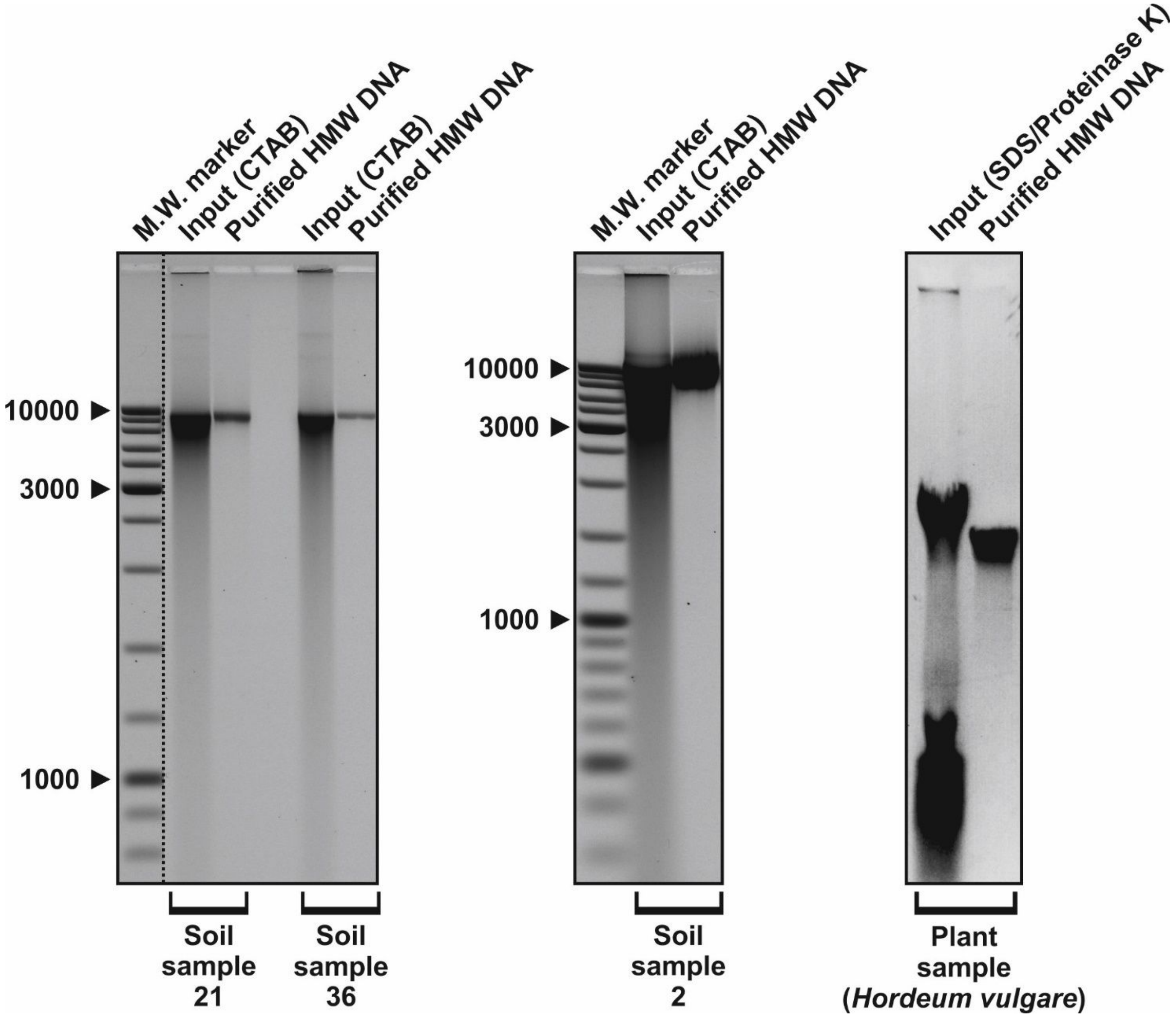

Figure S1. The proposed method consistently produces HMW DNA of high purity and integrity from complex soil and plant samples. Shown are negative images of ethidium bromide-stained agarose gels. Figure captions are as in **Figure 3**. Note the absence of fragmented DNA, low-molecular-weight nucleic acids (e.g., rRNA), and heavy covalent complexes in the purified HMW DNA samples. Soil samples were collected in Ylistaro, Finland, under the framework of the research project "Grasslands as a Carbon Sink" (https://www.luke.fi/en/projects/juurihiili).

Supplementary Tables

Table S1. Long-read sequencing report of soil samples processed using different DNA extraction methods.

<table>
<tr><th>Flow cell ID</th><th>Number of pores</th><th>Number of samples</th><th>Sample ID</th><th>Barcode ID</th><th>OUTLIERS Read length (kb) Outliers'?</th><th>Read length (kb)</th><th>Estimated reads</th><th>Base-called reads</th></tr>
<tr><td>FAS63057 (X1)</td><td>922</td><td>1</td><td>1 soil DNA purified with gel</td><td>1</td><td>27.75 - 28</td><td>2.78 k</td><td>5.55 Mb</td><td>4.78 Mb</td></tr>
<tr><td rowspan="2">FAS47629 (X2)</td><td rowspan="2">1477</td><td rowspan="2">2</td><td>2 soil DNA purified with gel</td><td>2</td><td rowspan="2">114 - 115</td><td rowspan="2">14.16 k</td><td rowspan="2">43.65 Mb</td><td rowspan="2">38.56 Mb</td></tr>
<tr><td>1_2 soil DNA purified with gel</td><td>3</td></tr>
<tr><td rowspan="3">FAV38481 (X3)</td><td rowspan="3">961</td><td rowspan="3">3</td><td>2_2 soil DNA purified with gel</td><td>4</td><td rowspan="3">63 - 63.5</td><td rowspan="3">8.74 k</td><td rowspan="3">22.43 Mb</td><td rowspan="3">20.15 Mb</td></tr>
<tr><td>3_2 soil DNA purified with gel</td><td>5</td></tr>
<tr><td>4_2 soil DNA purified with gel</td><td>6</td></tr>
<tr><td rowspan="2">FAU49011 (X5)</td><td rowspan="2">1464</td><td rowspan="2">2</td><td>21 soil DNA purified with the E.Z.N.A. soil DNA Extraction Kit</td><td>7</td><td rowspan="2">114 - 115</td><td rowspan="2">2.4 k</td><td rowspan="2">7.97 Mb</td><td rowspan="2">6.59 Mb</td></tr>
<tr><td>36 soil DNA purified with the E.Z.N.A. soil DNA Extraction Kit</td><td>8</td></tr>
</table>
